# Impact of Low-Starch Dietary Modifications on Faecal Microbiota Composition and Gastric Disease Scores in Performance Horses

**DOI:** 10.3390/ani15131908

**Published:** 2025-06-28

**Authors:** Jessica Irving, Violaine Pineau, Susanne Shultz, Fe ter Woort, Félicie Julien, Sandrine Lambey, Emmanuelle van Erck-Westergren

**Affiliations:** 1 School of Earth and Environmental Sciences, The University of Manchester, Manchester M13 9PT, UK; susanne.shultz@manchester.ac.uk; 2 Equine Sports Medicine Practice, 83 Avenue Beau Séjour, 1410 Waterloo, Belgium; fterwoort@esmp.be (F.t.W.); evanerck@esmp.be (E.v.E.-W.); 3 Lydia Becker Institute of Immunology and Inflammation, School of Biological Sciences, Faculty of Biology, Medicine & Health, Manchester M13 9PL, UK; 4 Manchester Environmental Research Institute, The University of Manchester, Manchester M13 9PL, UK; 5 Lambey SAS, Moulin des Prés, 71270 Torpes, France; f.julien@lambey.com (F.J.); s.lambey@lambey.com (S.L.)

**Keywords:** equine, microbiome, stomach, disease, diet, nutrition

## Abstract

Equine gastric disease (EGD) is a common condition in performance horses (*Equus caballus*), potentially compromising behaviour, performance, and welfare. EGD is often attributed to high-starch, high-sugar feeds and limited forage. Evidence for diet-induced changes on digestive microbiota is lacking. Nine elite showjumping horses were housed at the same performance yard with standardised diet and management throughout the study. Horses were transitioned from a high-sugar and -starch (31%) feed to a low-starch and -sugar (16.5%) concentrate feed. Gastroscopies, blood, and faecal samples were taken pre- and 12 weeks post-diet change. Squamous and glandular ulceration was blindly graded a posteriori using 0–4 scores and faecal microbiota profiled using 16S rRNA gene amplicon sequencing. Total (t_(1,8)_ = −6.17, *p* < 0.001; Pre: 4 [0–5], Post: 1 [0–2]), squamous (t_(1,8)_ = −5.32, *p* < 0.001; Pre: 1 [0–3], Post: 0 [0–1]), and glandular (t_(1,8)_ = −2.53, *p* = 0.04; Pre: 2.5 [0–4], Post: 0 [0–2]) disease improved following the introduction of a low-starch diet. Diet change did not impact microbiota communities (PERMANOVA: F_(1,16)_ = 1.37, *p* = 0.15, r^2^ = 0.08), but Firmicute to Bacteroidota (F/B) ratio reduced (t_(1,8)_ = −3.13, *p* = 0.01; Pre: 2.07 ± 0.21 vs. Post: 1.29 ± 0.14). Lower F/B ratios were associated with reduced total EGD scores (ChiSq_(1,17)_ = 3.83, *p* = 0.05). Low-starch diets did not influence faecal microbiota diversity but aided gastric disease healing and reduced F/B ratios in elite showjumpers during a competition season without medication.

## Simple Summary

Gastric ulcers are a common problem in performance horses and can affect their health, behaviour, and welfare. Ulcers are often linked to feeding practices involving high levels of starch and sugar and not enough fibre. Unfortunately, not all horses respond well to medication, so feed-based approaches to managing gastric ulcers alongside veterinary treatment are highly desirable. We examined how switching from a high- to low-starch diet affected gastric ulcers and the gut microbiome in elite showjumping horses. Over a 12 week period, we observed improvements in stomach health, with fewer and less severe ulcers following the diet change. Although we found no overall changes in gut bacteria communities, the balance between two major groups of bacteria shifted in a manner previously linked to improved gut health. These findings suggest that diet changes alone, without the use of anti-ulcer drugs, may improve gastric ulcer healing in performance horses.

## 1. Introduction

Equine gastric disease (EGD) is a prevalent and debilitating disease, encompassing lesions in the proximal squamous region (equine squamous gastric disease [ESGD]) and the distal glandular region (equine glandular gastric disease [EGGD]) of the stomach. EGD is prevalent in performance horses, affecting up to 100% of racehorses [1], 60% of eventers [2], and 70% of showjumpers [3]. Clinical signs are variable, but competition horses may present with subpar performance, reduced appetite and bodyweight [4]. Competing horses are typically provisioned with high-starch and -sugar feeds, alongside reduced quantities of long-stem forage, both established risk factors for ESGD development [5]. Risk factors for EGGD development are still unclear, but include stress, exercise intensity and frequency, high-starch diets [6], and reduced feeding frequency [7].

As hindgut fermenters, horses rely on a rich and diverse intestinal microbiota for fibre digestion, producing short-chain volatile fatty acids (VFAs) acetate, butyrate, and propionate, which supply up to 65% of the horse’s daily energy requirements [8,9]. In healthy horses, the faecal microbiota is dominated by Firmicutes, Bacteroidetes, and Proteobacteria [10,11,12]. However, disease, diet, and antimicrobial or pharmaceutical use can negatively impact microbiota composition and fermentation capacity. Starch-rich diets and intestinal disease reduce microbiota diversity and richness [13,14] and increase abundance of lactic acid-producing bacteria [15,16,17,18,19], reducing hindgut pH [13] and cellulolytic taxa abundance [20,21]. The Firmicute/Bacteroidetes (F/B) ratio has been proposed as a clinical indicator of gut dysbiosis in human patients [22], and high F/B ratios correspond with small and large intestinal colic in horses [23].

The high cost of medication, prescription requirements, and failure of clinical responses in many EGD cases [24] have encouraged investigations into nutritional supplements for preventing, or healing, gastric disease [25,26,27,28]. Altering feeding schedules successfully reduces EGD severity scores in unridden horses [29]; however, ad libitum access to long-stem forage has the greatest gastro-protective effect [30] by buffering gastric pH [31].

Understanding the influence of diet and feeding schedules on gut health in performance horses can contribute to the prevention of EGD and improve management and welfare in these populations. The objectives of the study were to determine if a low-starch feed, designed to protect gut health, would (1) reduce gastric disease severity and influence blood parameters, and (2) induce compositional changes in the digestive microbiota of competing showjumpers.

## 2. Materials and Methods

### 2.1. Study Population

Nine showjumping Warmbloods, involved in national and FEI (1* to 5*) competitions, stayed at the same yard before and throughout the study. Our inclusion criteria required horses to be between 4–16 years old, in active showjumping training and competition, receiving no medication, and with no overt signs of gastrointestinal disease or compromised health. Horse ages ranged from 4–13 years old, with an average bodyweight 604 ± 4.3 kg at initial assessment, and 605 ± 4.3 kg post-diet change. Their average body condition score (BCS) was 5 ± 0.3 pre- and 5 ± 0.2 post-diet change, measured on a standardised 9-point BCS scale [32]. Horses were housed in individual stables for 18 h per day, bedded on shavings, and allowed access to turnout in separate grass paddocks for four hours per day. In the twelve weeks preceding the study, and throughout the twelve weeks post-diet change period, the horses exercised six times per week and maintained their competition schedules and levels. Full details of individual horse BCS, bodyweight, age, and exercise and competition schedules twelve weeks prior to, and throughout, the study period are detailed in Table S1. Nine weeks after implementing the diet change, Horse 7 received a course of antibiotics for a respiratory tract infection between the two sampling points.

### 2.2. Diet Change

Dietary requirements were determined based on BCS, total bodyweight (BWT), and exercise level. Initial assessments occurred when the horses were fed a high-starch and -sugar (31%) pelleted concentrate diet (HS feed; Table 1). This had been the maintenance diet for the prior 18 months. Horses were transitioned onto a low-starch and -sugar (16.5%) concentrate feed (Regul Digest, Lambey SAS, Torpes, France; Table 1), for twelve weeks, before subsequent re-assessment. Regul Digest is a complete feed with dry alfalfa chaff and variable particulate sizes, marketed as supporting gastrointestinal health. Horses were gradually transitioned from the HS feed to the low-starch feed over seven days to minimise gastrointestinal disturbance, with the twelve-week assessment period commencing on the first day horses exclusively received the new feed. Pre-diet change, horses were fed the HS feed at three points during the day (09:00, 12:00, 17:00) with Omento Sport fibre blocks (Omento, Switzerland) at 21:00. Post-diet change, horses were fed Regul Digest at four timepoints (09:00, 12:00, 17:00, and 21:00). Horses were provided with hay at 1.6% to 2.4% BWT/day, divided into four meals. All horses received the same quantity of hay, fed at the same timepoints (09:00, 12:00, 17:00, and 21:00), before and throughout the study. Hay was steamed pre-feeding to reduce respirable allergens using a commercial hay steamer (Haygain^®^, London, UK). We provided water and salt licks ad libitum, and no other supplementary feeding. Concentrate and hay quantities were weighed (kg) prior to feeding. A control visit for weight and BCS assessment was conducted four weeks after study commencement and feed quantities were adjusted if necessary. Horses were not administered any anti-ulcer medication during the study.

### 2.3. Blood Collection

Venous blood was collected before and twelve weeks after changing the diet via jugular venipuncture into heparinized, plain, citrate, and EDTA tubes, and submitted for haematology and biochemistry at an external laboratory (Antech, Heppignies, Belgium).

### 2.4. Gastric Disease Scoring

Veterinarians performed gastroscopies at the initial assessment and twelve weeks post-diet change. Horses had feed withheld for 15–18 h and water for 2–3 h, and were sedated with 0.04 mg/kg romifidine (Sedivet^®^, Boehringer Ingelheim International GmbH, Ingelheim am Rhein, Germany), and the gastric mucosa was assessed using a flexible 3 m video-endoscope (VO320; Optomed^®^, Les Ulis, France). Gastric lesions were graded based on the score from Pineau et al. [33]. The total EGD score was calculated by adding the non-glandular and glandular lesion scores, both based on previously published scoring systems [30,34,35,36,37,38], with scores providing a qualitative severity rating from no pathology (NP) to moderately severe lesions (Table 2). After gastric mucosa assessment, we aspirated and collected 50 mL of gastric fluid using a sterile catheter inserted through the biopsy port of the endoscope, and collected rectal faecal samples whilst horses were sedated. We stored gastric aspirates and faecal samples in sample collection tubes (PowerBead Pro Tubes, Qiagen, Hilden, Germany) and sent them to an external microbiota sequencing company (EquiBiome^®^, Bangor, UK). Gastric disease was blindly scored by the two attending veterinarians (EVE and VP) a posteriori. Gastric disease scores pre- and post-diet change are shown as median [range].

### 2.5. Microbiota Sequencing

The commercial equine microbiota sequencing company EquiBiome^®^ performed faecal DNA extraction and rRNA gene sequencing. Faecal bacterial communities were profiled by 2 × 250 bp Illumina^®^ (San Diego, CA, USA) next-generation Miseq amplicon sequencing of the V3–V4 region of the 16S rRNA gene. We downloaded demultiplexed paired-end sequences, with non-biological nucleotides removed, from the EquiBiome^®^ Illumina^®^ BaseSpace Sequence Hub in FASTQ format for microbiota community analyses.

### 2.6. Microbiota Analyses

We performed all microbiota analyses on RStudio (v.4.3.1), using the dada2 package [39] (v.1.26) for initial filtering, trimming, and merging, sequence table construction, and chimera removal stages. We assigned taxonomy to amplicon sequence variants (ASVs) using the native Bayesian classifier database, Silva [40] (v.132). We used the DECIPHER package [41] (v.2.28.0) to align sequences for phylogenetic analyses, before using the phangorn [42] (v.2.11.1), and FastTree [43] (v.2.1.11) packages to construct maximum likelihood trees. All graphs were plotted using the ggplot2 package [44] (v.3.4.2).

We identified the core microbiota communities at the Operational Taxonomic Unit (OTU) level by determining OTUs shared across all horses pre- and post-diet change at ≥0.1% relative abundance, as previously described [45]. We determined the most abundant taxa at the phylum and family level from mean abundance measures before and after diet change. Proportion percentage changes of individual taxa pre- and post-diet change were calculated using the phylosmith [46] (v.1.0.7) and corncob [47] (v.0.3.2) packages.

Faecal microbiota alpha and beta diversity were computed using the phyloseq package [48] (v.1.44.0). We determined changes in alpha diversity metrics for richness (Shannon and Simpson’s diversity indices) using pairwise *t*-tests with Bonferroni correction. For beta diversity, we determined variation in individual microbiota phyla, families and class abundance, and faecal Firmicute to Bacteroidetes (F/B) ratio changes, between pre- and post-diet change using pairwise *t*-tests with Bonferroni correction. We additionally selected bacterial families reported to have nutritional amylolytic or cellulolytic functions [45], or involvement in lactic acid production [49], from previous reviews. Variation in relative abundance of amylolytic or cellulolytic groups between the two sampling timepoints was determined using pairwise *t*-tests with Bonferroni correction.

The packages microbiomeMarker [50] (v.1.6.0) and microbial (v.0.0.20) were used to plot Linear Discriminate Analysis (LDA) Effect Size (LeFSe) bacterial taxa changes in response to diet change. All data was log_10_ transformed, normalized using counts per million (CPM), with a Kruskal–Wallis cut-off of 0.05 and an LDA cut-off value of 4. Due to the small sample size of nine horses, we removed multiple comparisons from LeFSe analysis. We assessed changes in gut-specific functional groups in response to diet modification using the prokaryote database NJC19 [51] in the microeco package [52] (v.1.0.0), using a differential abundance test of traits percentage and random forest model, reporting MeanDecreaseGini scores, with multiple comparisons removed due to the small sample size.

### 2.7. Associations Between Microbiota Structure and Gut Health

We used Shapiro–Wilk tests to assess data normality. We determined the influence of diet change on total EGD, glandular, and squamous disease scores using pairwise *t*-tests with Bonferroni correction. We determined relationships between faecal F/B ratio and total EGD, glandular, and squamous scores using linear mixed models with Type III Wald tests, with gastric disease score as the response variable, F/B ratio as the explanatory variable, and horse as the random variable. Linear mixed models with Type III Wald tests assessed the relationship between F/B ratio and selected blood markers, with blood marker as the response variable, F/B ratio as the explanatory variable, and horse as the random variable. We built linear mixed models using the lme4 package [53] (v.1.1-27.1) with Type III Wald tests using the car package [54] (v.3.1-2).

### 2.8. Blood Parameter Responses to Diet Change

Blood biochemistry and haematology parameters were used to broadly screen the health status of the population pre- and post-diet change. As all horses were competitive showjumpers, we retained biochemistry markers of liver and muscle functioning gamma glutamyl-transferase (GGT), aspartate amino-transferase (AST), and creatinine kinase (CK) and the antioxidant marker vitamin E as variables of interest. We assessed changes in selected blood parameter concentrations at the two assessment timepoints using pairwise t-tests with Bonferroni correction. Mean blood marker concentrations (±standard error of the mean [SEM]) were calculated using the plotrix [55] (v.3.8-2) package. Vitamin E values post-diet change were not available for Horse 4; therefore, we excluded this horse from pre/post analysis for this marker.

### 2.9. Microbiota Community Composition and Gastric Disease Score

To determine the variation in beta dispersion within faecal microbiota in response to diet change, we calculated microbiota beta dispersion (PERMDISP) using the microeco package [52]. Intra-individual variation in faecal microbiota community structure was calculated using permutational multivariate analysis of variance (PERMANOVA), with 10,000 permutations, using the microeco package [52] and *adonis2*() in the vegan package [56] (v.2.5.7). Associations between faecal microbiota community structure and total EGD, glandular, and non-glandular scores were determined using PERMANOVA. We determined relationships between faecal microbiota communities and individual variables, including body condition score, sex, age, and the influence of the individual horse, using PERMANOVA.

We calculated principal component analyses (PCAs) using covariance matrices and associated eigenvalues and eigenvectors, produced using the maximum-likelihood phylogenetic tree and centred log ratio (clr) transformed values, and constructed using the microeco [52], microViz [57] (v.0.11.0), GUniFrac [58] (v.1.8), caret [59] (v.6.0-94), and ggplot2 [44] packages. PERMANOVA tests using *adonis2*() in the vegan package [56] were used to assess total dissimilarity of the faecal microbiota across diet change and EGD qualitative rating. We used taxa annotations on the PCA at phylum level to show sample clustering and similarity pre- and post-diet change in faecal microbiota, and associations between faecal microbiota community structure and total EGD rating.

## 3. Results

### 3.1. Blood Parameter Responses to Diet Change

Haematology parameters remained within normal limits pre- and post-diet change.

GGT (U/L), AST (UI/L), and CK (U/L) activity significantly decreased after diet change (Table 3). Antioxidant vitamin E (mg/L) concentration significantly increased after diet change, with two horses increasing from below the normal reference range prior to diet change. Additionally, GGT and AST activity, and vitamin E concentrations were associated with total EGD (Table 4), EGGD, and ESGD scores, and CK activity with EGD and ESGD scores in linear mixed models (Table 4).

### 3.2. Influence of Diet Change on Gastric Disease

**Figure 1:**
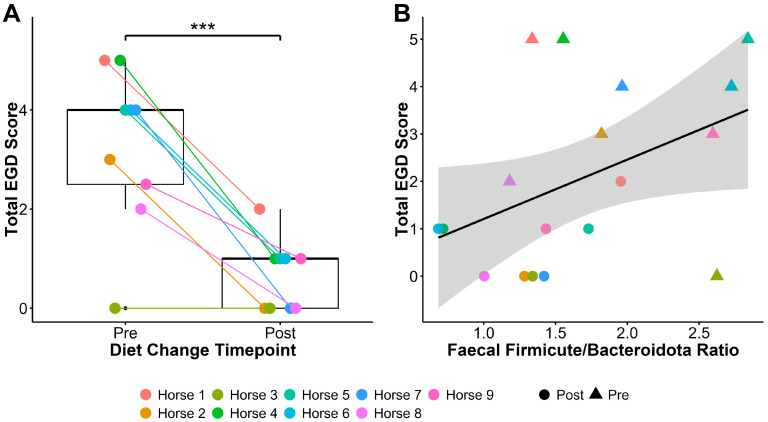
Total EGD score (**A**) significantly reduced after transitioning horses to a low-starch diet, and faecal Firmicute to Bacteroidota (F/B) ratio (**B**) was associated with reductions in total EGD score post-diet change. Boxplots in (**A**) show total EGD scores pre- and post-diet change: middle lines represent the median; the top and bottom of the box represent the 3rd and 1st quartiles, respectively; box height indicates the interquartile range (IQR); and whiskers extend to the furthest values within 1.5 × IQR. Plot (**B**) shows the relationship between F/B ratios and total EGD score, with triangles indicating pre-diet change, and circles indicating post-diet change samples. The solid line represents the fitted linear regression, and the shaded area shows the standard error around the regression line. *** = *p* < 0.001.

Glandular, squamous, and total gastric disease scores of all nine horses reduced between the two sampling time points. Total EGD score reduced from 4 [0–5] to 1 [0–2] (Pairwise t_(1,8)_ = −6.17, *p* = 0.0002; Figure 1A), EGGD score from 2 [0–4] to 0 [0–2] (t_(1,8)_ = −2.53, *p* = 0.04), and ESGD score from 1 [0–3] to 0 [0–1] (t_(1,8)_ = −5.32, *p* = 0.0007).

### 3.3. Gastric and Faecal Microbiota Phylogenetic Sequencing

After chimeras were removed, 1,311,714 paired-end sequences were retained as high-quality sequences from 18 samples. Faecal samples had an average read number of 16,226 ± 712.3 and sequence reads were similar pre- and post-diet change. A total of 27,162 ASVs were identified across all samples. Across faecal samples, 99.5% of reads were classified at phylum, 72.6% at family, and 45% at genus level. Gastric fluid samples collected during gastroscopy were additionally sent for sequencing; however, due to poor sequence quality, these were excluded from final analyses.

### 3.4. Core Microbiota

**Figure 2:**
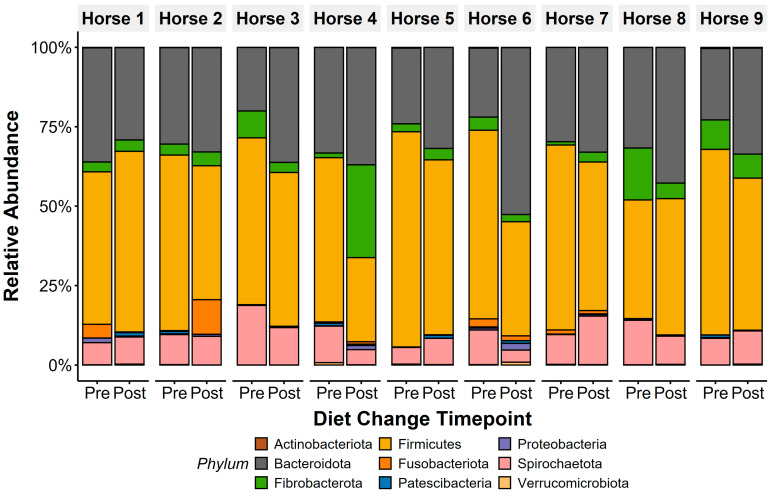
Faecal microbiota composition of all horses was dominated by anaerobic bacterial phyla, with Firmicutes and Bacteroidota presenting the highest abundance in both sample types. Other anaerobic taxa, including Fibrobacterota, Spirochaetota, and Fusobacteriota, were additionally identified pre- and post-diet changes. Only phyla with >0.1% mean relative abundance are displayed.

The core microbiota community at the Operational Taxonomic Unit (OTU) level was determined by identifying OTUs shared across all horses in the study at ≥0.1% relative abundance, as previously described [45]. Faecal microbiota were heavily dominated by the phylum Firmicutes (pre: 54.2 ± 2.8%, post: 44.6 ± 3.1%; Figure 2), with Bacteroidota as the second most abundant phylum (pre: 27.6 ± 1.9%, post: 36.4 ± 2.4%). Families from the phyla Firmicutes (*Lachnospiraceae*; pre: 20.2 ± 2.5%, post: 10.2 ± 1.4%; *Erysipelatoclostridiaceae*: pre: 7.6 ± 1.6%, post: 8.7 ± 1.2%), Spirochaetota (*Spirochaetaceae*: pre: 10.5 ± 1.3%, post: 8.9 ± 1.2%), Bacteroidota (*Rikenellaceae*: pre: 8.5 ± 1.2%, post: 10.1 ± 1.1%), and Fibrobacterota (*Fibrobacteraceae*: pre: 5.5 ± 1.7%, post: 6.8 ± 2.9%) were highly abundant in faecal microbiota.

### 3.5. Alpha Diversity Measures

**Figure 3:**
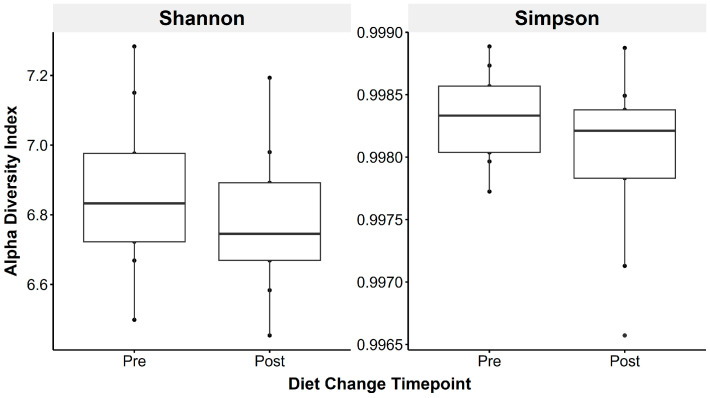
Faecal microbiota alpha diversity metrics were not significantly altered by transitioning horses to a low-starch diet, using the Shannon (**left**) and Simpson (**right**) indices, reflecting species richness and evenness.

Faecal microbiota species richness did not change following the introduction of a low-starch diet (Shannon: t_(1,8)_ = 0.83, *p* = 0.43; Simpson: t_(1,8)_ = 1.49, *p* = 0.18; Figure 3).

### 3.6. Beta Diversity Measures

#### 3.6.1. Changes in Microbiota Composition

**Figure 4:**
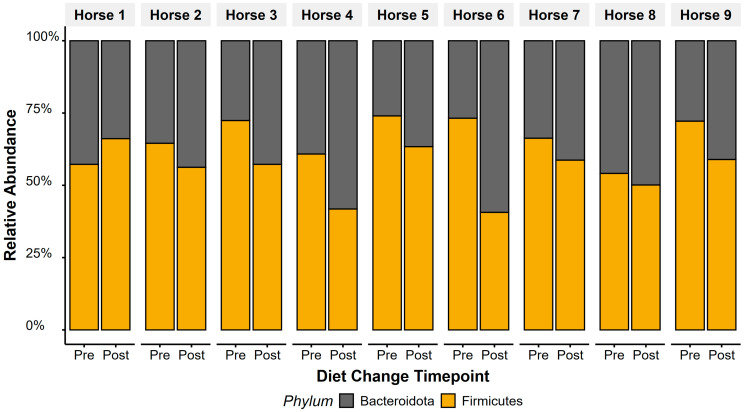
Twelve weeks after transitioning to a low-starch diet, relative abundance of Firmicutes decreased and Bacteroidetes increased, reflecting significant responses in faecal F/B ratios to diet change. Average faecal F/B ratios dropped from 2.07 (±0.21) to 1.29 (±0.14).

Bacteroidota relative abundance increased (t_(1,8)_ = 2.5, *p* = 0.04) and Firmicutes decreased (t_(1,8)_ = −2.5, *p* = 0.037) in faecal microbiota after diet change (Figure 4). Overall, this led to a decrease in F/B ratios following the diet change (t_(1,8)_ = −3.13, *p* = 0.01), with faecal F/B ratio dropping from a ratio of 2.07 (±0.21) to 1.29 (±0.14). Horse 1 showed opposing patterns to Horses 2–9, showing an increase in F/B ratios after diet change.

#### 3.6.2. Changes in Functional Groups

The relative abundance of *Lachnospiraceae* (t_(1,8)_ = −4.73, *p* = 0.002), but not *Lactobacillaceae* (t_(1,8)_ = −2.13, *p* = 0.07), reduced after diet change. Grouped lactic acid-producing families, including *Lactobacillaceae*, *Streptococcaceae*, *Spirochaetaceae,* and *Rikenellaceae* decreased in abundance after diet change (t_(1,80)_ = −4.32, *p* = 0.0004). Grouped amylolytic families, including *Streptococcaceae*, *Succinivibrionaceae*, *Lactobacillus*, and *Enterococcaceae*, similarly reduced in abundance post-diet change (t_(1,35)_ = −2.07, *p* = 0.05). Diet change did not influence the relative abundance of grouped cellulolytic families, including *Ruminococcaceae*, *Fibrobacteraceae*, *Lachnospiraceae*, *Prevotellaceae*, *Eubacteriaceae*, *Clostridiaceae,* and *Acidaminococcaceae* (t_(1,62)_ = 1.01, *p* = 0.32) and the mucin-producing phyla *Verrucomicrobiota* did not respond to diet change (t_(1,8)_ = 0.38, *p* = 0.71). Linear Discriminate Analysis (LDA) Effect Size (LEfSe) (Figure S1) shows decreases in Firmicutes phyla (LDA: 5.03, *p* = 0.02), and enrichment in Bacteroidota phyla (LDA: 4.97, *p* = 0.01), in faecal microbiota from pre- to post-diet changes. Decreases in abundance of other taxa in response to diet change include *Lachnospiraceae* (LDA: 5.7, *p* = 0.004) and *Oscillospiraceae* (LDA: 4.97, *p* = 0.04). Taxa abundance enrichment below the phylum level post-diet change were all below the LDA cut-off value.

Functional potential of faecal microbiota community gut taxa pathways was predicted using the prokaryote database NJC19 [51]. There was no significant enrichment in digestion pathways after diet change; however, non-significant decreases in H_2_O_2_ production were identified in faeces post-diet change (MeanDecreaseGini: 0.25; *p* = 0.09).

#### 3.6.3. Associations Between Microbiota Structure and Gut Health

Irrespective of timing, lower Firmicute to Bacteriodota ratios (F/B ratios) were associated with lower total EGD (linear mixed models [LMM]: ChiSq_(1,17)_ = 3.83, *p* = 0.05; Figure 1B) and EGGD (ChiSq_(1,17)_ = 6.38, *p* = 0.01) scores, but not ESGD scores (ChiSq_(1,17)_ = 0.8, *p* = 0.4). Faecal F/B ratio was additionally associated with elevated vitamin E concentration (LMM: ChiSq_(1,17)_ = 6.88, *p* = 0.009), and reduced GGT activity (ChiSq_(1,17)_ = 6.68, *p* = 0.01) and CK (ChiSq_(1,17)_ = 7.31, *p* = 0.007).

#### 3.6.4. Microbiota Community Composition and Gastric Ulceration Score

Diet change did not lead to significant changes in intra-individual (PERMANOVA; F_(1,16)_ = 1.37, *p* = 0.2, r^2^ = 0.08; Figure S2A) faecal microbiota community structure, despite observed variations between individual horses. Microbiota beta dispersion did not vary within faecal microbiota communities in response to diet change (PERMDISP; F_(1,16)_ = 0.02, *p* = 0.8). Gastric disease severity scoring categories pre- and post-diet change were not associated with intra-individual (PERMANOVA; F_(6,11)_ = 1.06, *p* = 0.4, r^2^ = 0.06) differences in faecal microbiota community structure (Figure S2B).

Faecal microbiota communities were not associated with total EGD score (PERMANOVA; F_(6,11)_ = 0.9, *p* = 0.6, r^2^ = 0.05), EGGD (F_(5,12)_ = 0.94, *p* = 0.5, r^2^ = 0.06), or ESGD (F_(5,12)_ = 0.74, *p* = 0.7, r^2^ = 0.04) scores. Faecal microbiota composition was not associated with other variables, including individual horse (F_(8,9)_ = 0.77, *p* = 0.9, r^2^ = 0.41), body condition score (F_(5,10)_ = 0.87, *p* = 0.6, r^2^ = 0.05), sex (F_(2,13)_ = 0.85, *p* = 0.7, r^2^ = 0.10), and age (F_(4,11)_ = 0.84, *p* = 0.8, r^2^ = 0.21).

## 4. Discussion

Within our population of high-level showjumpers, switching from a high-starch (29%) to a low-starch (11%) concentrate feed supported gastric disease healing during a competitive season without medication. Although overall microbiota diversity or composition remained unchanged, faecal Firmicute to Bacteroidetes (F/B) ratios reduced, aligning closely with ratios previously reported in healthy horses [23].

Gastric disease is prevalent in high-level performance horses, with risk factors including breed [60], competition schedules [3], and transportation stress [61]. In our cohort, 89% of horses presented with gastric lesions ≥ Grade 2 severity at study onset, higher than previously reported in showjumpers [3], yet lower than racehorses in training [1]. Although pharmaceuticals are the primary treatment for gastric ulceration, spontaneous healing without treatment is not well documented [62], and dietary supplement efficacy remains inconsistent [25,26,27,28]. Dietary management, particularly providing low-starch diets, has shown efficacy at preventing ESGD [63] and EGGD recurrence [35].

As performance level, exercise intensity and pasture turnout were unchanged in the twelve weeks before and after diet change (Table S1), high dietary starch likely contributed to initial gastric disease development. Although the low-starch feed contained a marginally higher sugar content (Table 1), simple sugars can promote *Lactobacillus* colonisation in the equine glandular stomach [35], a mechanism that aids ulcer healing in rats [64]. Variable particulate sizes and short-stem alfalfa in the tested feed may have reduced mechanical mucosal injury during peristalsis [65]. Alfalfa’s high calcium and protein content also buffer gastric pH [66], reducing EGGD severity and incidence [35]. While we cannot definitively isolate which feed component, whether particulate size, macronutrient (starch, fat, sugar), or micronutrient composition was most beneficial, gastric ulcer scores improved after twelve weeks of feeding, and the same horses showed no change in exercise performance [33]. These findings warrant further targeted investigations into how specific macronutrients, micronutrients, and physical feed characteristics exert gastroprotective [35,65,66] or gastro-injurious [67,68,69,70] effects in equid gastric disease.

Although regular grazing is considered protective against gastric disease, our horse cohort had four hours of grass paddock access, plus daily roughage at 1.6–2.4% of bodyweight in the twelve weeks preceding, and throughout the study. Indeed, broodmares at pasture still develop gastric lesions [71], whereas management changes alongside ad libitum roughage feeding assist lesion healing, even without omeprazole [72]. Despite the time at grass remaining standardised before and after diet change, we did not quantify seasonal fluctuations in grass nutrient content; however, we saw no microbiota shifts typically associated with seasonal grazing changes [73]. Future studies should integrate longitudinal microbiota sequencing, repeated gastroscopies, and analysis of grass non-structural and water-soluble carbohydrate levels to clarify how seasonal pasture variation affects equid gastric health.

Faecal microbiota were dominated by Firmicutes (pre: 54%, post: 45%) and Bacteroidota (pre: 28%, post: 36%), mirroring profiles in Thoroughbred racehorses [74,75] and Arabian endurance horses [76]. In contrast, non-performance horses exhibit higher Bacteroidota abundance [10], indicating performance horses may exhibit distinct faecal microbiota compared to convalescing, retired, or leisure horses. Despite differences in gastric physiology, reduced Bacteroidetes abundance is associated with gastric disease in humans [77], similar to our observation of lower Bacteroidota pre-diet change when lesion scores were highest. Faecal microbiota communities, however, are unlikely to reflect proximal intestine or gastric conditions, and our observed F/B shifts likely reflect dietary alterations more than EGD severity. Nevertheless, these parallels suggest, as in human athletes [78,79,80,81], exercise capacity and diet may shape distinct microbiota signatures in performance horses.

Alpha-diversity and β-diversity metrics remained stable between sampling timepoints. As all horses were deemed clinically healthy throughout, pronounced microbiota dysbiosis was not expected, and the lack of diversity shifts post-diet change was anticipated. However, the F/B ratio decreased after diet change (pre: 2.07 ± 0.21, post: 1.29 ± 0.14), approaching values reported in healthy horses [23]. Elevated Firmicutes and F/B ratios have been linked to gastrointestinal disease [23], doxycycline treatment [82], and obesity [83,84] in humans and horses. Although no standardised healthy F/B threshold exists in equids [84,85,86,87], our findings indicate faecal F/B ratios show potential as a non-invasive microbiota marker of gastrointestinal health.

While nearly all exhibited reduced Firmicutes abundance and F/B ratios after diet change, Horse 1 showed the opposite pattern. Horse 7 received doxycycline for a respiratory infection at week 9, and showed no microbiota community changes. However, by the final sampling timepoint, the faecal microbiome may have stabilized [86], masking any doxycycline-associated impact. The cause of Horse 1’s divergent response remains unclear. Future studies with larger cohorts and more frequent sampling points are necessary to elucidate intra-individual microbiota variability across time.

Before diet change, lactic acid-producing taxa (*Lachnospiraceae*, *Lactobacillaceae,* and *Lachnospiraceae*) and amylolytic taxa (*Streptococcaceae*, *Lactobacillus,* and *Enterococcus*) were more abundant. Overgrowth of these families lowers intestinal pH [8], disrupts microbiota balance [88], and can precipitate hindgut acidosis, laminitis, and colic [23]. *Oscillospiraceae* was also elevated pre-diet change, but its function in the equine gut remains unclear, despite links to obesity and metabolic disorders in humans [89]. These compositional shifts likely reflect decreased starch fermentation; however, further investigations (e.g., faecal pH, metabolite profiling) are required to determine the roles of lactic acid-producing, amylolytic, and cellulolytic taxa in equid digestive health.

We monitored liver and muscle enzymes (CK, GGT, AST) and antioxidant status (vitamin E) due to their potential performance benefits. All blood enzymes and vitamin E remained within normal limits at both timepoints (Table 3), but CK, AST, and GGT activity decreased, whereas vitamin E concentrations increased after diet change. In our horses, higher EGD, ESGD, and EGGD scores associated with higher CK, AST, and GGT, yet lower vitamin E. These patterns align with equid reports linking gastric disease to high systemic CK [90] and GGT [91] and high salivary GGT, CK [92], and AST [93], alongside rat studies showing lower blood GGT [94] during ulcer healing. Additionally, lower GGT has been linked to improved feed digestibility [95,96] and improved training adaptations [97]. However, given our sample size constraints, we cannot discern whether these blood enzyme and antioxidant changes stem from gastric healing or dietary provision.

This study has some limitations: the EGD quantitative grading system, unmeasured grass nutrients, lack of control group, small sample size, and two-timepoint sampling may have restricted some inferences. We implemented an ordinal EGD scoring method [33], combining glandular and squamous scores [30,34,35,36,37,38], to capture lesion extent and distribution, and expected mucosal healing responses of erythematous, haemorrhagic, fibrinosuppurative lesions (Table 2), while preserving resolution and inter-observer reliability [30,37,98]. Although this approach may introduce measurement error, we minimised this by using two blinded, experienced veterinarians for lesion scoring. Secondly, we attempted gastric fluid microbiota sequencing, but low read counts necessitated exclusion from analysis. Recent successful sequencing (e.g., [17]) indicates tissue and gastric juice sampling may be required. Thirdly, we did not quantify seasonal variation in grass nutrients, which may have contributed to gastric healing. Fourthly, our small sample size, with only two sampling timepoints, limits some conclusions due to the potential for sampling bias and low statistical power. Finally, although our longitudinal, within-individual design controls for individual variability, the lack of an additional control group limits our ability to account for time-related confounders or broader population-level changes that may have influenced outcomes independently of the diet change.

## 5. Conclusions

We show a commercial low-starch concentrate feed assisted in reducing gastric disease scores and improving faecal F/B ratios in competing performance horses without conventional anti-ulcer medication. Although initial lesion severity was mild, reflecting naturally occurring ulcers in this population, these same horses exhibited fewer ridden pain-associated behaviours after diet change [33]. While causal inference is limited by our small cohort, these results underscore the critical role of dietary composition in equine gastric functionality and lesion healing. Moreover, they highlight the need for deeper investigations into gut microbiota dynamics during exercise and competition in high-level performance horses.

## Figures and Tables

**Table 1:** Nutrient composition of supplementary and long-stem forage portions of the diet provided throughout the length of the study (per kg feed), the overall nutrient intake per day (per kg feed), and the average ingested nutrients per day (g/kg body weight [BWT]) pre- and post-diet change.

Nutritional Analyses	Pre		Post	Hay *	Overall Nutrition (per Day)		Ingested Nutrients (g/kg BWT per Day)	
	Omento Sport	HS Feed	Regul Digest		Pre	Post	Pre	Post
DE (MJ/kg)	7.4	10.8	11.9	5.65	23.85	17.55	0.52	0.41
Crude protein (%)	9	11	11.5	10.45	30.45	21.95	6.69	5.13
Crude fats (%)	4	3	6.4	2.63	9.63	9.03	2.12	2.11
Crude fibre (%)	19.1	14.5	12	27.44	60.64	39.44	13.32	9.22
Ash (%)	8.2	7.0	9.0	8.5	23.7	17.5	5.21	4.09
Sodium (%)	0.3	0.2	0.4	-	0.5	0.4	0.03	0.03
Starch (%)	13	29	11	-	42	11	2.72	0.87
Sugars (%)	-	2.5	5.5	5.1	7.6	10.6	1.67	2.48
Calcium (%)	0.83	1	1.2	0.65	2.48	1.85	0.55	0.43
Phosphorous (%)	0.27	0.5	0.6	0.26	1.03	0.86	0.23	0.20

* The hay nutritional composition remained the same throughout the length of the study. DE = digestible energy; MJ = megajoules; BWT = total body weight.

**Table 2:** The equine gastric disease quantitative grading method utilized [33] adapted from previously published scoring systems [30,34,35,36,37,38], with a maximum severity score of 8.

Severity Score	Area of Gastric Mucosa Assessed		Total EGD Severity Grading *
	Non-Glandular Lesion Score	Glandular Lesion Score	
0	No pathology (NP)	No pathology (NP)	No pathology (NP)
1	Single, small, multifocal lesions	Mild to moderate focal erythematous areas (gastritis)	Mild (grading 1–2)
2	Large single or extensive superficial lesions	Moderate to severe focal to multifocal erythematous extensive areas	Moderate (grading 3–4)
3	Deep ulcers present	Erythematous areas with focal fibrinosuppurative lesions	Moderate (grading 5–6)
4	Extensive areas of deep ulceration, with bleeding ulcers	Erythematous areas with focal fibrinosuppurative and haemorrhagic lesions	Severe (grading 7–8)

* Total EGD score was determined by adding the severity score of non-glandular and glandular lesions together.

**Table 3:** Pairwise *t*-tests indicated blood liver and muscle enzyme markers reduced after transitioning to a low-starch diet, whereas circulating vitamin E concentrations increased after diet change.

	Mean (±SEM) Blood Marker Concentrations		Normal Reference Range	Mean Difference ± Confidence Interval (CI)	t	df	*p*
	**Pre**	**Post**					
Gamma Glutamyl Transferase [GGT] (U/L)	41.56 ± 5.04	16.89 ± 2.19	10–40	−24.67 ± 10.22	−5.57	8	<0.001
Aspartate aminotransferase (AST) (UI/L)	581.56 ± 43.57	319.33 ± 13.40	160–400	−199.23 ± 45.58	−6.26	8	0.0002
Creatine Kinase [CK] (U/L)	333.89 ± 44.45	274.78 ± 29.39	60–330	−59.11 ± 42.02	−3.24	8	0.01
Vitamin E (mg/L)	2.89 ± 0.28	3.84 ± 0.52	2.2–7.7	1.04 ± 0.74	3.33	7	0.01

SEM = standard error; df = degrees of freedom; t = t statistic; *p* = *p*-value.

**Table 4:** Linear mixed models were used to determine relationships between total (EGD), squamous (ESGD), and glandular (EGGD) gastric disease scores and liver and muscle enzyme markers and circulating vitamin E concentrations.

	Total EGD				EGGD				ESGD			
	t	df	ChiSq	*p*	t	df	ChiSq	*p*	t	df	ChiSq	*p*
Gamma Glutamyl Transferase [GGT] (U/L)	5.31	17	28.19	0.0001	4.65	17	21.66	0.0001	3.28	17	10.74	0.001
Aspartate aminotransferase (AST) (UI/L)	4.25	17	18.08	<0.0001	5.39	17	29.06	<0.0001	2.23	17	4.95	0.03
Creatine Kinase [CK] (U/L)	2.22	17	4.94	0.03	2.31	17	5.33	0.02	1.90	17	3.62	0.06
Vitamin E (mg/L)	−2.83	16	8.02	0.005	−2.26	16	6.85	0.009	−2.08	16	6.18	0.04

Models were built with the blood marker (e.g. Vitamin E) as the response variable, ulcer score/ type (e.g. EGD score, EGGD score, or ESGD score) as the independent variable, and individual horse was included as a random variable. Positive t-scores indicate positive associations between total EGD score and the blood parameter, and negative t-scores indicate negative associations. t = t statistic; df = degrees of freedom; ChiSq = Chi-squared distribution; *p* = *p*-value.

## Data Availability

If the paper is accepted for publication, all associated sequence data will be uploaded to GenBank for data sharing. Further data presented in the study is available on request to the corresponding authors.

## Supplementary Materials

The following supporting information can be downloaded at: https://www.mdpi.com/article/10.3390/ani15131908/s1, Table S1: Competition level, number of competitions, and ridden exercise frequency and level remained standardised in the twelve weeks preceding the diet change, and in the twelve weeks after diet change; Figure S1. LEfSe analysis was used to identify changes in microbiota taxa across phylum, family, order, and genus levels after transitioning horses to a low-starch diet; Figure S2. Principal component analysis (PCA) produced at the phylum level, representing impacts of transitioning horses to a low-starch diet. Reference [32] is cited in the Supplementary Materials.

## Abbreviations

The following abbreviations are used in this manuscript:

AST	Aspartate amino-transferase
ASV	Amplicon sequence variants
BCS	Body condition score
BWT	Body weight
ChiSq	Chi-squared distribution (χ^2^)
CK	Creatine kinase
CI	Confidence interval
CLR	Centred log ratio
CPM	Counts per million
DE	Digestible energy
DNA	Deoxyribonucleic acid
ECEIM	European College of Equine Internal Medicine
EDTA	Ethylenediaminetetraacetic acid
EGD	Equine Gastric Disease
EGGD	Equine Glandular Gastric Disease
EGUS	Equine Gastric Ulcer Syndrome
ESGD	Equine Squamous Gastric Disease
F/B Ratio	Firmicute to Bacteroidetes Ratio
FEI	Fédération Équestre Internationale
GGT	Gamma glutamyl-transferase
H_2_O_2_	Hydrogen peroxide
HK	Hyperkeratosis
IQR	Interquartile range
LDA	Linear discriminate analysis
LEfSe	Linear discriminate analysis effect size
LMM	Linear mixed model
MJ	Megajoules
NP	No pathology
NSC	Non-structural carbohydrate
OTU	Operational taxonomic unit
PERMANOVA	Permutational multivariate analysis of variance
PCA	Principal component analysis
rRNA	Ribosomal RNA
SEM	Standard error of the mean
VFA	Volatile fatty acids
WSC	Water-soluble carbohydrate
