# Supplementary material for: Impact of Low-Starch Dietary Modifications on Faecal Microbiota Composition and Gastric Disease Scores in Performance Horses
Source: Animals (Basel). 2025 Jun 28;15(13):1908. doi: 10.3390/ani15131908 (PMC12248642; doi:10.3390/ani15131908)
Supplement: Supplementary file 1 [file animals-15-01908-s001.zip › animals-3667538-supplementary.pdf]

Table S1. Competition level, number of competitions, and ridden exercise frequency and level remained standardised in the twelve weeks preceding the diet change, and in the twelve weeks after diet change.

Horse	12 weeks pre-diet change				12 weeks post-diet change			
	BWT (kg)	BCS	Competition level	# times ridden per week	BWT (kg)	BCS	Competition level	# times ridden per week
1	610	6+	CSI 1*	6	608	6	CSI 1*	6
2	642	5+	CSI 5*	6	646	5	CSI 5*	6
3	598	6+	CSI 2*	6	606	6-	CSI 2*	6
4	682	5+	CSI 2*	6	676	5	CSI 2*	6
5	576	4	National, 95 cm	6	606	4+	National, 95 cm	6
6	606	6-	CSI YH	6	590	5+	CSI YH	6
7	582	5	National, 115 cm	6	592	5-	National, 115 cm	6
8	592	4+	National, 95 cm	6	582	4	National, 95 cm	6
9	550	6	National, 105 cm	6	540	5+	National, 105 cm	6

BWT = total bodyweight (kg); BCS = measured on a 1–9 validated scoring system [32]; CSI = Concours de Saut International (International Jumping Competition); CSH YH = Concours de Saut International (International Jumping Competition) for Young Horses; CSI 1* = maximum height 140 cm; CSI 2* = maximum height 145 cm; CSI 5* = maximum height 160 cm;

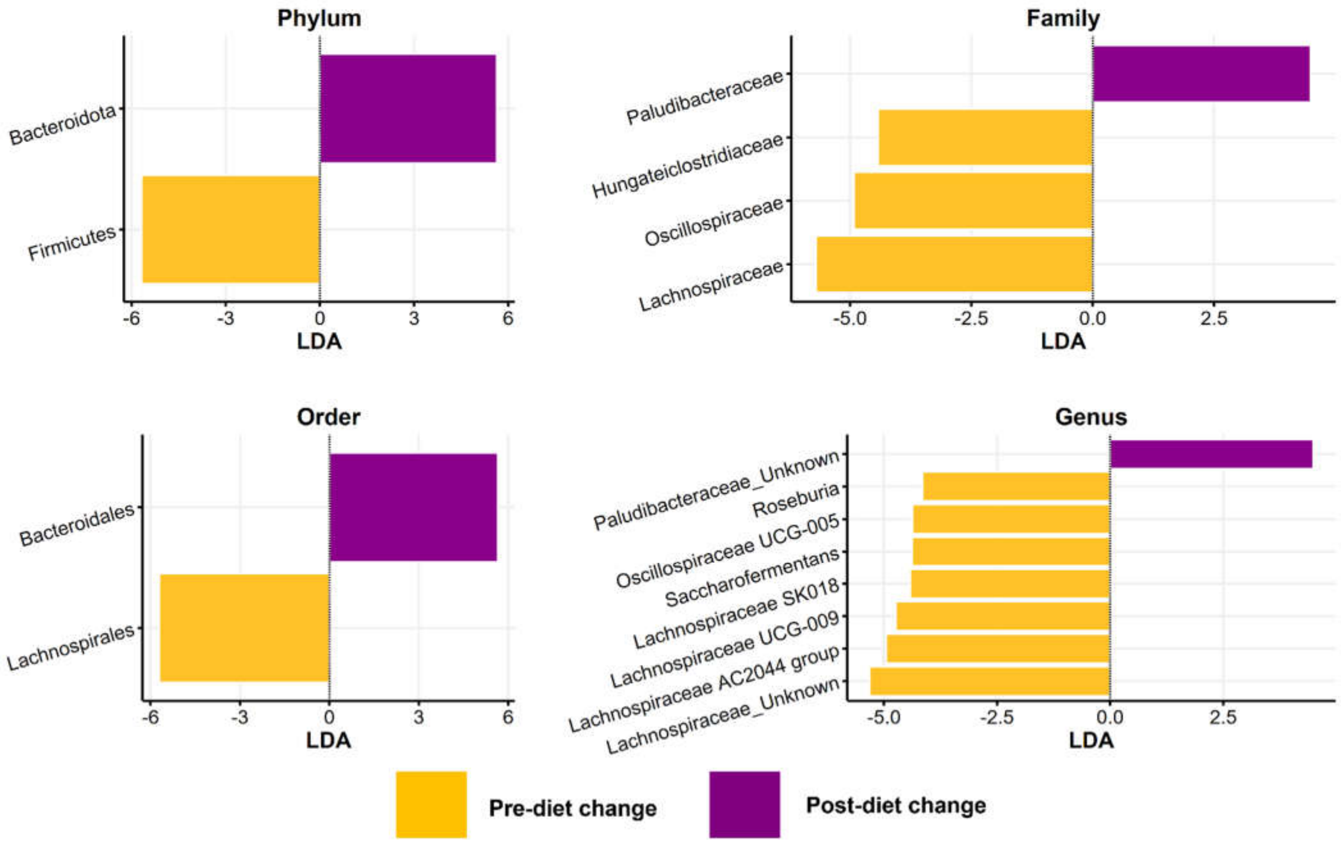

Figure S1. LEfSe analysis was used to identify changes in microbiota taxa across phylum, family, order, and genus levels after transitioning horses to a low-starch diet. We subsequently used linear discriminate analyses (LDA) to determine the effect size of differentially abundant taxa. The *Bacteroidota* phylum, *Bacteroidales* order, and *Paludibacteraceae* families were enriched in faecal microbiota composition after diet change.

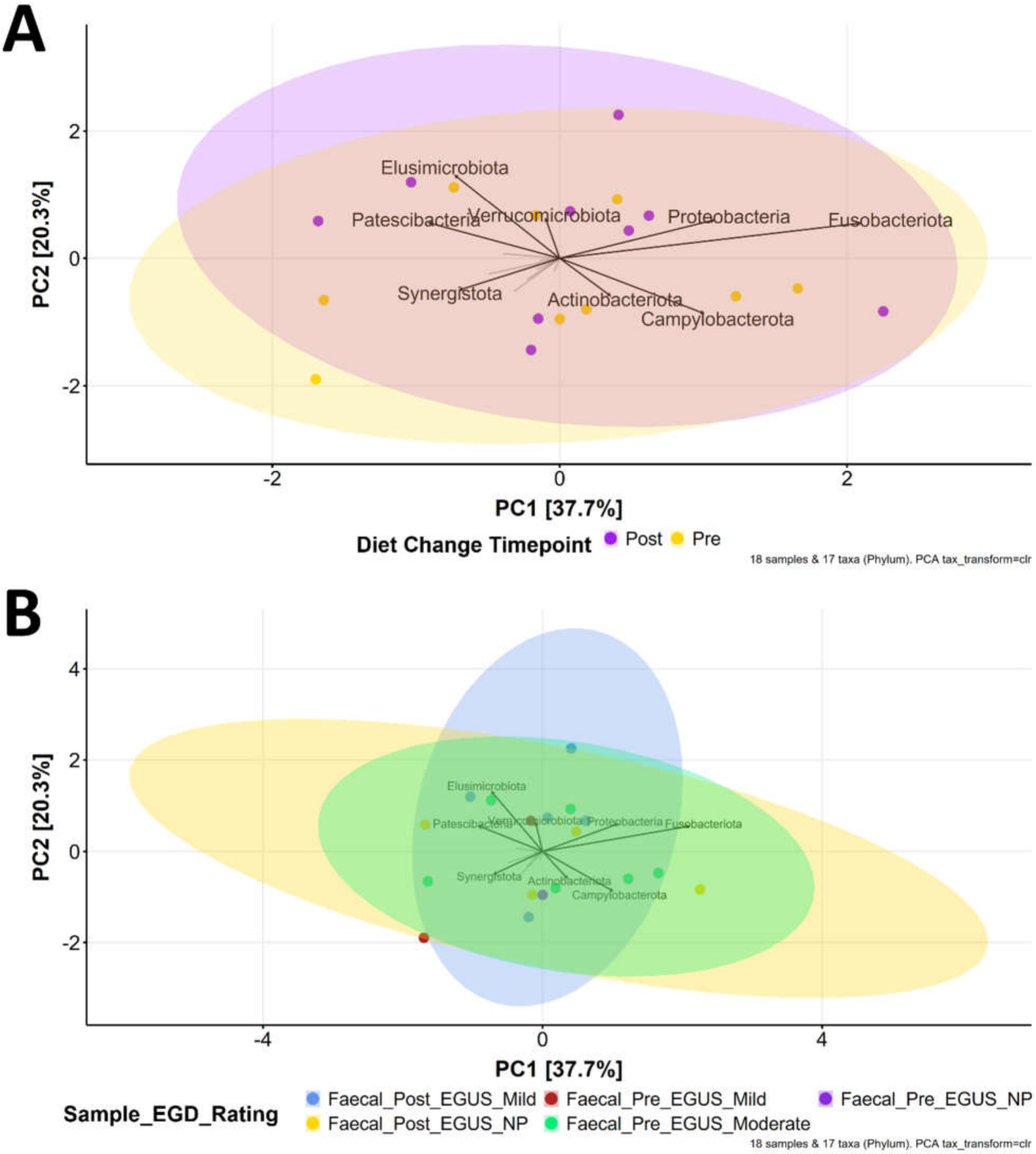

Figure S2. Principal component analysis (PCA) reflecting the minimal microbiota shifts in the top 8 most abundant phyla after transitioning horses to a low-starch diet. Diet change did not influence faecal microbiome community structure (A), and faecal microbiome communities were not associated with total gastric disease scoring severities (Table 2) pre- and post-diet change (B). EGUS = Equine Gastric Ulcer Syndrome, NP = no pathology.
